# Healthy Dietary Intake Behavior Potentially Modifies the Negative Effect of COVID-19 Lockdown on Depression: A Hospital and Health Center Survey

**DOI:** 10.3389/fnut.2020.581043

**Published:** 2020-11-16

**Authors:** Khue M. Pham, Linh V. Pham, Dung T. Phan, Tien V. Tran, Hoang C. Nguyen, Minh H. Nguyen, Huu C. Nguyen, Tung H. Ha, Hung K. Dao, Phuoc B. Nguyen, Manh V. Trinh, Thinh V. Do, Hung Q. Nguyen, Thao T. P. Nguyen, Nhan P. T. Nguyen, Cuong Q. Tran, Khanh V. Tran, Trang T. Duong, Lam V. Nguyen, Thao T. Do, Tam T. Vo, Binh N. Do, Thai H. Duong, Thu T. M. Pham, Thuy T. Le, Ngoc T. Do, Hoai T. T. Nguyen, Thuy T. T. Mai, Dung T. Ha, Huong T. M. Ngo, Kien T. Nguyen, Shwu-Huey Yang, Jane C.-J. Chao, Tuyen Van Duong

**Affiliations:** ^1^Faculty of Public Health, Hai Phong University of Medicine and Pharmacy, Hai Phong, Vietnam; ^2^President Office, Hai Phong University of Medicine and Pharmacy, Hai Phong, Vietnam; ^3^Department of Pulmonary and Cardiovascular Diseases, Hai Phong University of Medicine and Pharmacy Hospital, Hai Phong, Vietnam; ^4^Director Office, Hai Phong University of Medicine and Pharmacy Hospital, Hai Phong, Vietnam; ^5^Faculty of Nursing, Hanoi University of Business and Technology, Hanoi, Vietnam; ^6^Nursing Office, Viet Duc University Hospital, Hanoi, Vietnam; ^7^Department of Infectious Diseases, Vietnam Military Medical University, Hanoi, Vietnam; ^8^Director Office, Military Hospital 103, Hanoi, Vietnam; ^9^Director Office, Thai Nguyen National Hospital, Thai Nguyen, Vietnam; ^10^President Office, Thai Nguyen University of Medicine and Pharmacy, Thai Nguyen, Vietnam; ^11^International Master/Ph.D. Program in Medicine, Taipei Medical University, Taipei, Taiwan; ^12^Director Office, E Hospital, Hanoi, Vietnam; ^13^Department of Thoracic and Cardiovascular Surgery, E Hospital, Hanoi, Vietnam; ^14^Director Office, General Hospital of Agricultural, Hanoi, Vietnam; ^15^Director Office, Bac Ninh Obstetrics and Pediatrics Hospital, Bac Ninh, Vietnam; ^16^Director Office, Kien an Hospital, Hai Phong, Vietnam; ^17^Director Office, Quang Ninh General Hospital, Quang Ninh, Vietnam; ^18^Director Office, Bai Chay Hospital, Quang Ninh, Vietnam; ^19^Director Office, Quang Ninh Obstetrics and Pediatrics Hospital, Quang Ninh, Vietnam; ^20^Health Management Training Institute, Hue University of Medicine and Pharmacy, Hue, Vietnam; ^21^Department of Health Economics, Corvinus University of Budapest, Budapest, Hungary; ^22^General Planning Department, Da Nang Oncology Hospital, Da Nang, Vietnam; ^23^Director Office, Thu Duc District Health Center, Ho Chi Minh City, Vietnam; ^24^Faculty of Health, Mekong University, Vinh Long, Vietnam; ^25^Director Office, Hospital District 2, Ho Chi Minh City, Vietnam; ^26^Nursing Office, Tan Phu District Hospital, Ho Chi Minh City, Vietnam; ^27^President Office, Can Tho University of Medicine and Pharmacy, Can Tho, Vietnam; ^28^Aesthetic Plastic Surgery & Skin Care Center, Can Tho University of Medicine and Pharmacy Hospital, Can Tho, Vietnam; ^29^Department of Oral Pathology and Periodontology, Faculty of Odonto-Stomatology, Can Tho University of Medicine and Pharmacy, Can Tho, Vietnam; ^30^Director Office, Trieu Phong District Health Center, Quang Tri, Vietnam; ^31^Division of Military Science, Military Hospital 103, Hanoi, Vietnam; ^32^Department of Internal Medicine, Thai Nguyen University of Medicine and Pharmacy, Thai Nguyen, Vietnam; ^33^Faculty of Medical Laboratory Science, Da Nang University of Medical Technology and Pharmacy, Da Nang, Vietnam; ^34^President Office, Da Nang University of Medical Technology and Pharmacy, Da Nang, Vietnam; ^35^Nursing Office, E Hospital, Hanoi, Vietnam; ^36^Training and Direction of Healthcare Activity Center, Kien an Hospital, Hai Phong, Vietnam; ^37^Nursing Office, Quang Ninh General Hospital, Quang Ninh, Vietnam; ^38^Nursing Office, Bai Chay Hospital, Quang Ninh, Vietnam; ^39^Nursing Office, Quang Ninh Obstetric and Pediatric Hospital, Quang Ninh, Vietnam; ^40^Department of Health Education, Faculty of Social Sciences, Behavior and Health Education, Hanoi University of Public Health, Hanoi, Vietnam; ^41^School of Nutrition and Health Sciences, Taipei Medical University, Taipei, Taiwan; ^42^Nutrition Research Center, Taipei Medical University Hospital, Taipei, Taiwan; ^43^Research Center of Geriatric Nutrition, Taipei Medical University, Taipei, Taiwan; ^44^Master Program in Global Health and Development, College of Public Health, Taipei Medical University, Taipei, Taiwan

**Keywords:** COVID-19, coronavirus, lockdown, healthy eating, psychological, physical activity, comorbidity, obesity

## Abstract

**Background:** The COVID-19 pandemic causes a huge burden for affected countries. Several public health interventions were applied to contain the infection. However, the pandemic itself and the lockdown measure negatively influence people's lifestyles and psychological health.

**Purpose:** To explore determinants of healthy dietary intake and depression, and examine the interaction between healthy dietary intake and COVID-19 lockdown on depression.

**Methods:** A cross-sectional study was conducted at 18 hospitals and health centers from February 14 to May 31, 2020. Data of 8,291 outpatients were collected including patients' characteristics, clinical parameters, health literacy, healthy dietary intake (using the healthy eating score, HES), other health-related behaviors, and depression (using the patient health questionnaire, PHQ). Depression was defined as PHQ score ≥ 10.

**Results:** Protective factors of healthy dietary intake and depression were higher education, better medication payment ability, higher social status, more physical activity, and higher health literacy, whereas older age, ever married, own business or other types of occupation, lockdown, suspected COVID-19 symptoms, and comorbidity were associated with lower HES scores and a higher depression likelihood. Besides, overweight/obesity and alcohol drinking were associated with lower HES scores. As compared with patients not under lockdown and with lowest HES score, those who were under lockdown and with lowest HES score had 10.6 times higher depression likelihood (odds ratio, OR, 10.60; 95% CI 6.88, 16.32; *p* < 0.001), whereas people with higher HES score had 15% lower depression likelihood (OR 0.85; 95% CI 0.82, 0.89; *p* < 0.001).

**Conclusions:** Healthy dietary intake and depression were determined by several sociodemographic, clinical, and behavioral factors. Lockdown measure affects people's dietary intake behavior and depression. Importantly, healthy dietary intake potentially modifies the negative effect of lockdown on depression.

## Introduction

The COVID-19 pandemic is caused by the severe acute respiratory syndrome coronavirus 2 (SARS-CoV-2), which sets the whole world in unprecedented challenges (1–6). It creates a huge burden, in terms of socioeconomic effects (7), morbidity, and mortality (8, 9). Infections and deaths are dramatically increasing in all the affected countries (10). Multidisciplinary and multidimensional approaches are required to contain the pandemic (11–13). In the situation of unavailable effective treatments and vaccination, social and behavioral changes are highly recommended to control the massive global health crisis (14, 15).

Among COVID-19 management strategies, healthy diet and nutrition show potential impacts on immune system and health outcomes (16–18). A diversified and balanced diet can improve the immune response to viral infection (19). Healthy foods have been found as a potential therapy to enhance immunity, to improve the acute respiratory symptoms and health outcomes which may help to protect people during the COVID-19 pandemic (20). Some food groups (e.g., fruits and vegetables, fish and fish oils) and key nutrients (e.g., fiber, vitamins A, B, C, D, and E, selenium, iron, copper, zinc) have shown the benefit for protecting against viral infection (17, 21, 22). Adequate intake of relevant nutrients can help to reduce inflammation and oxidative stress, which further strengthens the immune system of individuals during the COVID-19 pandemic (22, 23).

The COVID-19–induced lockdown or home confinement measure was applied in many countries including Vietnam (24). This measure is a necessary public health approach to protect people from virus infection. However, it has undesirable consequences (25), e.g., negative impacts on psychological consequences (26, 27), eating behavior, and changes in dietary habits (28–31). Fortunately, healthy diet has potential benefits to reduce the risk of severity (32) and complications of COVID-19 (33). People with a better diet quality intake had a lower risk of depression (34, 35). Moreover, assessment of dietary intake behavior is critically important for identifying the comprehensive approach to manage COVID-19 (36) and indicating the sustainable food intake during the lockdown (37). The healthy eating score (HES-5) is a short, simple, and valid tool to quickly assess the overall diet quality which is comparable with the 2015 health eating index (38). The HES-5 has an advantage of timely and easy assessment of healthy dietary intake behavior in the time of COVID-19 pandemic.

Therefore, we investigated the associated factors of healthy dietary intake behavior and depression, as well as examined the interaction of COVID-19 lockdown and healthy dietary intake on depression among people who visited outpatient departments from 18 hospitals and health centers across Vietnam.

## Methods

### Study Design and Settings

A cross-sectional study was conducted from February 14 to May 31, 2020. Study duration in each hospital/health center was 7–10 days. The study participants were consecutively recruited at outpatient departments (OPDs) from 15 hospitals and three health centers across Vietnam. The study sites were conveniently selected, including 10 hospitals and one health center in the North, one hospital and one health center in the Center, and four hospitals and one health center in the South.

### Study Sample

Participants were those who visited the OPDs of selected hospitals and health centers. The recruited participants were those aged 18 to 85 years, understood Vietnamese, and without emergency conditions. After excluding 60 individuals with age <18 years (26 cases), more than 85 years (19 cases), and incomplete survey (15 cases), a total sample of 8,291 outpatients were analyzed. Participants in studied hospitals and health centers are presented in Table 1.

**Table 1:** Participants in studied hospitals and health centers.

**Geographic location**	**Hospital/health center**	**Studied participants**
**North**		
Ha Noi city	Military Hospital 103	1,028
	E hospital	183
	General Hospital of Agricultural	300
Thai Nguyen province	Thai Nguyen National Hospital	469
Bac Ninh city	Bac Ninh Obstetrics and Pediatrics Hospital	500
Hai Phong city	Hai Phong University of Medicine and Pharmacy Hospital	982
	Kien An Hospital	492
	Kien Thuy District Health Center	484
Quang Ninh province	Quang Ninh General Hospital	309
	Bai Chay Hospital	364
	Quang Ninh Obstetrics and Pediatrics Hospital	280
**Center**		
Quang Tri province	Trieu Phong District Health Center	495
Da Nang city	Da Nang Oncology Hospital	421
**South**		
Ho Chi Minh city	Thu Duc District Hospital	489
	Thu Duc District Health Center	497
	Hospital District 2	248
	Tan Phu District Hospital	242
Can Tho city	Can Tho University Of Medicine and Pharmacy Hospital	508
Total		8,291

### Assessments and Measurements

#### Participants' Characteristics

Participants self-reported their information, including age (years), gender (women vs. men), marital status (never married vs. ever married), educational attainment (illiterate/elementary, junior high school, senior high school, college/university or higher), occupation (employed, own business, and others), ability to pay for medication (very difficult to very easy), and social status (patients placed themselves into the society regarding education, career, and salary, at three levels from low, middle to high). Vietnam had applied the nationwide lockdown measure from April 1 to 22, 2020 (24, 39). Therefore, the lockdown was defined for patients who took the survey during that period.

#### Clinical Parameters

Patients were asked to report their body height (cm) and weight (kg). Body mass index (BMI, kg/m^2^) was calculated. The suspected COVID-19 symptoms (S-COVID-19-S) were assessed (40), including common symptoms (fever, cough, dyspnea) and less common symptoms (myalgia, fatigue, sputum production, confusion, headache, sore throat, rhinorrhea, chest pain, hemoptysis, diarrhea, and nausea/vomiting). Patients were classified as having S-COVID-19-S if they had any of those symptoms. Items of the Charlson Comorbidity Index were used to screen for comorbidity (41).

#### Health-Related Behaviors

Patients reported their current behaviors as compared with before the pandemic, including smoking status (never/stop/less vs. unchanged or more), drinking status (never/stop/less vs. unchanged or more), and physical activities (never/stop/less vs. unchanged or more).

#### Health Literacy

The short-form health literacy questionnaire with 12 items (HLS-SF12) was used to assess health literacy (HL). The tool was validated and used in Asian countries (42) including Vietnam (43–46). Patients were asked to rate their perceived difficulty of each item based on 4-point Likert scales from 1 = “very difficult” to 4 = “very easy.” The overall score was standardized to an index ranged from 0 to 50, with higher score presenting better HL, using the formula (1):

(1) $$\begin{array}{l} Index=\left(Mean-1\right)\;\times \left(\frac{50}{3}\right) \end{array}$$

where *Index* is the specific index score calculated, *Mean* is the mean of 12 items for each individual, 1 is the minimal possible value of the mean (leading to a minimum index value of 0), 3 is the range of the mean, and 50 is the chosen maximum value of HL index.

#### Health Dietary Intake Behavior

The 5-item healthy eating score (HES-5) was used to assess healthy dietary intake behavior. HES-5 was validated and used in previous studies (38, 47). The utilization of HES-5 was comparable with the 2015 health eating index and quickly assesses the overall diet quality (38). The tool is expected to be useful for assessing the healthy dietary intake behavior during the sensitive period of the pandemic. The questionnaire was translated into Vietnamese by researchers. The content was then validated by an expert panel (28 medical doctors, 7 nurses, 9 nutrition and public health professionals). The expert panel suggested using the rating and the scoring of the original scale. The unidimensional construct was expressed with all five items loaded on one component (factor loadings ranged from 0.63 to 0.75), which explained 49.43% of the variance. The tool was showed with adequate convergent validity (item–scale correlation ranged from 0.57 to 0.73), satisfactory reliability (Cronbach's alpha of 0.74), and without floor or ceiling effects (Supplementary Table 1). Participants were asked about how often did they eat/drink fruits, vegetables, whole grains, dairy, and fish over the last 30 days. The rating scale was from 0 = “Rarely or never,” 1 = “1–2 times per week,” 2 = “3–6 times per week,” 3 = “once per day,” 4 = “twice per day,” to 5 = “3 or more times per day.” The total score of healthy dietary intake (HDI-score, or HES) ranged from 0 to 25, with the higher score indicating the better healthy eating behavior.

#### Depression

The patient health questionnaire with 9 items (PHQ-9) was used to assess depression. PHQ-9 is a screening tool that helps clinicians in making the diagnosis of depression, quantifying depression symptom, and monitoring the severity (48). This tool was used in Vietnam (45). Patients were asked about how often they have been bothered by nine symptoms during the last 2 weeks on the scale from 0 (not at all), 1 (several days), 2 (more than half the days), to 3 (nearly every day). The overall PHQ-9 score ranges from 0 to 27. Patients were classified as having depression if their PHQ score ≥10 (48).

### Data Collection Procedure

Before the data collection, we provided research assistants (doctors, nurses, and medical students) a 4 h training session on data collection. Research assistants also received the infection control training from each health facility, e.g., using masks, washing hands, and physical distancing according to guidelines of the Centers for Disease Control and Prevention (49), World Health Organization (50), and Vietnam Ministry of Health (51).

Research assistants contacted and asked patients who visited the OPDs for voluntary participation. The OPD visitors were consecutively invited to the survey. The consent form was obtained from qualified patients before administering the survey. The survey took place during the waiting time, before and/or after physical examination. At the early stage of the pandemic, face-to-face interviews were conducted. At the peak stage of the pandemic, self-administered questionnaires were used via an online version (QR code provided at each OPD) or printed version. It took about 20–30 min to complete survey questionnaires. Finally, data were confidentially analyzed by researchers.

### Ethical Consideration

The study was reviewed and approved by each participating hospital, and the Institutional Ethical Review Committee of Hanoi University of Public Health, Vietnam (IRB No. 029/2020/YTCC-HD3 for the first period from February 14 to March 31, 2020; and IRB No. 133/2020/YTCC-HD3 for the second period from April 1 to May 31, 2020).

### Statistical Analysis

First, distributions of studied variables were explored using the χ^2^ test and one-way ANOVA test appropriately. Second, associated factors of healthy dietary intake behavior (HES) and depression (PHQ) were examined using linear regression models and logistic regression models, respectively. To minimize residual effects of confounders on the associations, factors associated with HES or PHQ at *p* < 0.20 in the bivariate model were selected into the multivariate model (52). To avoid the multicollinearity in the multivariate models, the correlations of factors were tested using Spearman correlation. If the moderate or high correlations exist, a representative factor was selected to final models. Finally, the interaction analysis was conducted to examine the potential mental health benefits of healthy dietary intake behavior. Data were analyzed using the IBM SPSS version 20.0 (IBM, Armonk, NY, USA). The significance level was set at a *p* < 0.05.

## Results

### Participants' Characteristics

Mean values of age, health literacy, and healthy eating score (HES) were 43.6 ± 16.9, 28.1 ± 9.4, and 11.9 ± 4.6, respectively. Proportions of people who participated during the lockdown measure and with depression (PHQ ≥ 10) were 28.7% (2,376/8,291) and 12.5% (1,033/8,291), respectively. The HES was varied by different categories of age, gender, marital status, education, occupation, ability to pay for medication, social status, lockdown, S-COVID-19-S, BMI, comorbidity, smoking, drinking, and physical activity (*p* < 0.001), whereas the prevalence of depression was varied by different categories of age, marital status, education, occupation, ability to pay for medication, social status, lockdown, comorbidity (*p* < 0.001), and BMI (*p* = 0.028; Table 2).

**Table 2:** Participants' characteristics, healthy dietary intake behavior, and depression.

**Variables**	**Overall (*N* = 8,291)**	**HES (*N* = 8,291)**		**PHQ <10 (*N* = 7,258)**	**PHQ ≥ 10 (*N* = 1,033)**	
	***N* (%)**	**Mean ± SD**	p^*^	***N* (%)**	***N* (%)**	p^**^
Age, years			<0.001			<0.001
18–39	3,955 (47.7)	12.6 ± 4.7		3,688 (50.8)	267 (25.8)	
40–59	2,473 (29.8)	11.5 ± 4.5		2,220 (30.6)	253 (24.5)	
60–85	1,863 (22.5)	11.3 ± 4.4		1,350 (18.6)	513 (49.7)	
Gender			<0.001			0.906
Women	4,890 (53)	12.1 ± 4.6		4,279 (59.0)	611 (59.1)	
Men	3,401 (41)	11.7 ± 4.6		2,979 (41.0)	422 (40.9)	
Marital status			<0.001			<0.001
Never married	1,635 (19.8)	12.4 ± 4.5		1,496 (20.7)	139 (13.5)	
Ever married	6,628 (80.2)	11.8 ± 4.6		5,734 (79.3)	894 (86.5)	
Education attainment			<0.001			<0.001
Elementary school or illiterate	593 (7.2)	11.3 ± 4.7		480 (6.6)	113 (10.9)	
Junior high school	1,630 (19.7)	11.1 ± 4.3		1,431 (19.8)	199 (19.3)	
Senior high school	2,277 (27.5)	11.8 ± 4.4		1,995 (27.5)	282 (27.3)	
College/university or higher	3,776 (45.6)	12.5 ± 4.8		3,337 (46.1)	439 (42.5)	
Occupation			<0.001			<0.001
Employed	2,390 (28.9)	12.2 ± 4.7		2,149 (29.7)	241 (23.3)	
Own business	3,044 (36.8)	11.7 ± 4.6		2,709 (37.4)	335 (32.4)	
Others	2,843 (34.3)	12.1 ± 4.5		2,386 (32.9)	457 (44.2)	
Ability to pay for medication			<0.001			<0.001
Very or fairly difficult	4,475 (54)	11.5 ± 4.7		3,710 (51.2)	765 (74.1)	
Very or fairly easy	3,805 (46)	12.4 ± 4.4		3,537 (48.8)	268 (25.9)	
Social status			<0.001			<0.001
Low	1,403 (16.9)	10.9 ± 4.7		1,187 (16.4)	216 (20.9)	
Middle or high	6,879 (83.1)	12.2 ± 4.5		6,062 (83.6)	817 (79.1)	
Lockdown measure			<0.001			<0.001
No	5,915 (71.3)	12.4 ± 4.5		5,428 (74.8)	487 (47.1)	
Yes	2,376 (28.7)	10.9 ± 4.6		1,830 (25.2)	546 (52.9)	
S-COVID-19-S^a^			<0.001			<0.001
No	5162 (62.3)	12.7 ± 4.7		4,827 (66.5)	335 (32.4)	
Yes	3129 (37.7)	11.0 ± 4.6		2,431 (33.5)	698 (67.6)	
BMI, kg/m^2^			0.026			0.028
Underweight (BMI <18.5)	783 (9.5)	12.3 ± 4.7		709 (9.8)	74 (7.2)	
Normal weight (18.5 ≤ BMI <25.0)	6,518 (78.8)	11.9 ± 4.6		5,685 (78.4)	833 (81.0)	
Overweight/obese (BMI ≥ 25.0)	974 (11.8)	11.7 ± 4.6		853 (11.8)	121 (11.8)	
Comorbidity			<0.001			<0.001
None	6,415 (77.5)	12.5 ± 4.6		5,877 (81.1)	538 (52.1)	
One	1,458 (17.6)	10.4 ± 4.1		1,132 (15.6)	326 (31.6)	
Two or more	409 (4.9)	8.7 ± 3.2		241 (3.3)	168 (16.3)	
Smoking^b^			<0.001			0.867
Never, stopped, or smoke less	7,541 (91.0)	12.1 ± 4.6		6,600 (90.9)	941 (91.1)	
Unchanged or smoke more	750 (9.0)	10.6 ± 4.6		658 (9.1)	92 (8.9)	
Drinking alcohol^b^			<0.001			0.680
Never, stopped, or drink less	7,044 (85.1)	12.1 ± 4.6		6,178 (85.1)	866 (84.7)	
Unchanged or drink more	1,235 (14.9)	11.1 ± 4.6		1,078 (14.9)	157 (15.3)	
Physical activity^b^			<0.001			<0.001
Never, stopped, or exercise less	2,778 (33.6)	11.6 ± 4.9		2,190 (30.3)	588 (57.1)	
Unchanged or exercise more	5,480 (66.4)	12.1 ± 4.4		5,038 (69.7)	442 (42.9)	
HL index, 1-score increment	28.1 ± 9.4			28.7 ± 9.3	24.1 ± 9.6	<0.001^*^
HES, 1-score increment	11.9 ± 4.6			12.1 ± 4.6	10.9 ± 4.6	<0.001^*^

HES, healthy eating score; PHQ, patient health questionnaire; S-COVID-19-S, suspected corona virus disease-2019 symptoms; BMI, body mass index; HL, health literacy.

* Result of one-way ANOVA test.

** Result of χ^2^ test.

a The suspected COVID-19 symptoms including common symptom (fever, cough, dyspnea), less common symptom (myalgia, fatigue, sputum production, confusion, headache, sore throat, rhinorrhea, chest pain, hemoptysis, diarrhea, and nausea/vomiting).

b *People were asked whether their health-related behaviors are getting worse, better, or unchanged during COVID-19 pandemic as compared with those before the pandemic*.

### Associated Factors of Healthy Dietary Intake

In bivariate analysis, patients with lower HES were those with older age, being male, ever married, having own business, during the lockdown period, with S-COVID-19-S, underlying comorbidity, and smoking and drinking at unchanged or more level (*p* < 0.001). In contrast, patients with higher HES were those with higher educational attainment, better ability to pay for medication, higher social status, doing physical activity at unchanged or more level, and higher health literacy (*p* < 0.05; Table 3). Correlations among covariates were checked to eliminate the multicollinearity. Moderate correlations were found between age and marital status (rho = 0.38), education (rho = −0.42), comorbidity (rho = 0.31), and health literacy (rho = −0.32); between S-COVID-19-S and comorbidity (rho = 0.31); between lockdown measure and physical activity (rho = −0.38); and between smoking and drinking (rho = 0.45; Supplementary Table 2). Therefore, age, gender, occupation, ability to pay for medication, social status, lockdown, S-COVID-19-S, BMI, and drinking alcohol were selected to multivariate models. Results showed that as compared with counterparts, people with lower HES were those with older age (regression coefficient, B, −0.81, 95% CI −1.03, −0.58, *p* < 0.001 for age 40–59 years; and B, −0.70, 95% CI −0.95, −0.44, *p* < 0.001 for age 60–85 years), having own business (B,; 95% CI −0.53, −0.04; *p* = 0.021), during the lockdown period (B, −1.35; 95% CI −1.57, −1.13; *p* < 0.001), with S-COVID-19-S (B, −1.14; 95% CI −1.35, −0.94; *p* < 0.001), being overweight/obese (B, −0.34; 95% CI −0.64, −0.04; *p* = 0.025), and drinking alcohol at unchanged or more level (B, −1.29; 95% CI −1.57, −1.00; *p* < 0.001; Table 3). On the other hand, people with higher HES were those with better ability to pay for medication (B, 0.26; 95% CI 0.05, 0.47; *p* = 0.016) and higher social status (B, 0.92; 95% CI 0.65, 1.19; *p* < 0.001; Table 3).

**Table 3:** Associated factors of healthy dietary intake behavior via linear regression analysis (*N* = 8,291).

**Variables**	**HES**			
	**Bivariate**		**Multivariate**	
	**B (95% CI)**	***p***	**B (95% CI)**	***p***
Age, years				
18–39	0.00		0.00	
40–59	−1.09 (−1.32, −0.86)	<0.001	−0.81 (−1.03, −0.58)	<0.001
60–85	−1.27 (−1.52, −1.02)	<0.001	−0.70 (−0.95, −0.44)	<0.001
Gender				
Women	0.00		0.00	
Men	−0.37 (−0.57, −0.17)	<0.001	−0.04 (−0.24, 0.17)	0.710
Marital status				
Never married	0.00			
Ever married	−0.56 (−0.81, −0.31)	<0.001		
Education attainment				
Elementary school or illiterate	0.00			
Junior high school	−0.16 (−0.59, 0.27)	0.460		
Senior high school	0.54 (0.13, 0.96)	0.010		
College/university or higher	1.16 (0.77, 1.56)	<0.001		
Occupation				
Employed	0.00		0.00	
Own business	−0.51 (−0.76, −0.27)	<0.001	−0.28 (−0.53, −0.04)	0.021
Others	−0.12 (−0.37, 0.13)	0.363	0.02 (−0.23, 0.27)	0.878
Ability to pay for medication				
Very or fairly difficult	0.00		0.00	
Very or fairly easy	0.94 (0.74, 1.14)	<0.001	0.26 (0.05, 0.47)	0.016
Social status				
Low	0.00		0.00	
Middle or high	1.31 (1.05, 1.57)	<0.001	0.92 (0.65, 1.19)	<0.001
Lockdown measure				
No	0.00		0.00	
Yes	−1.51 (−1.72, −1.29)	<0.001	−1.35 (−1.57, −1.13)	<0.001
S-COVID-19-S^a^				
No	0.00		0.00	
Yes	−1.46 (−1.67, −1.26)	<0.001	−1.14 (−1.35, −0.94)	<0.001
BMI, kg/m^2^				
Underweight (BMI <18.5)	0.33 (−0.01, 0.67)	0.059	0.14 (−0.19, 0.47)	0.410
Normal weight (18.5 ≤ BMI <25.0)	0.00		0.00	
Overweight/obese (BMI ≥ 25.0)	−0.27 (−0.58, 0.04)	0.091	−0.34 (−0.64, −0.04)	0.025
Comorbidity				
None	0.00			
One	−2.15 (−2.40, −1.89)	<0.001		
Two or more	−3.86 (−4.30, −3.41)	<0.001		
Smoking^b^				
Never, stopped, or smoke less	0.00			
Unchanged or smoke more	−1.48 (−1.82, −1.13)	<0.001		
Drinking alcohol^b^				
Never, stopped, or drink less	0.00		0.00	
Unchanged or drink more	−0.99 (−1.27, −0.71)	<0.001	−1.29 (−1.57, −1.00)	<0.001
Physical activity^b^				
Never, stopped, or exercise less	0.00			
Unchanged or exercise more	0.54 (0.33, 0.75)	<0.001		
HL index, 1-score increment	0.10 (0.09, 0.11)	<0.001		

HES, healthy eating score; B, regression coefficient; PHQ, patient health questionnaire; S-COVID-19-S, suspected corona virus disease-2019 symptoms; BMI, body mass index; HL, health literacy.

a The suspected COVID-19 symptoms including common symptom (fever, cough, dyspnea), less common symptom (myalgia, fatigue, sputum production, confusion, headache, sore throat, rhinorrhea, chest pain, hemoptysis, diarrhea, and nausea/vomiting).

b *People were asked whether their health-related behaviors are getting worse, better, or unchanged during COVID-19 pandemic as compared with those before the pandemic*.

### Associated Factors of Depression

In bivariate analysis, odds of depression were significantly higher in older people, those ever married, other types of occupation, in lockdown period, with S-COVID-19-S, and underlying comorbidity as compared with their counterparts (*p* < 0.001). Odds of depression were significantly lower in people with higher education, better ability to pay for medication, higher social status, being underweight, doing physical activity at unchanged or more level, higher health literacy, and higher HES as compared with their counterparts (*p* < 0.01). To avoid multicollinearity, age, gender, occupation, ability to pay for medication, social status, lockdown measure, S-COVID-19-S, BMI, comorbidity, physical activity, and HES were included in multivariate models. The results showed that people with higher odds of depression were those with older age (odds ratio, OR, 1.33, 95% CI 1.10, 1.60, *p* = 0.004 for age 40–59 years; OR 3.03, 95% CI 2.52, 3.64, *p* < 0.001 for age 60–85 years) as compared with age 18–39 years, other type of occupation (OR 1.27; 95% CI 1.05, 1.54; *p* = 0.013) as compared with employed group, during lockdown (OR 1.85; 95% CI 1.56, 2.18; *p* < 0.001) as compared with not during the lockdown period, with S-COVID-19-S (OR 2.40; 95% CI 2.05, 2.81; *p* < 0.001) as compared with those without S-COVID-19-S, and those with comorbidity (OR 1.51, 95% CI 1.26, 1.80, *p* < 0.001; OR 2.19, 95% CI 1.68, 2.85, *p* < 0.001) as compared with those without chronic conditions. In contrast, people with lower odds of depression were those with better ability to pay for medication (OR 0.66; 95% CI 0.56, 0.78; *p* < 0.001) and doing physical activity at unchanged or more level (OR 0.62; 95% CI 0.53, 0.73; *p* < 0.001; Table 4).

**Table 4:** Associated factors of depression via logistic regression analysis (*N* = 8,291).

**Variables**	**Depression (PHQ ≥ 10)**			
	**Bivariate**		**Multivariate**	
	**OR (95% CI)**	***p***	**OR (95% CI)**	***p***
Age, years				
18–39	1.00		1.00	
40–59	1.57 (1.32, 1.88)	<0.001	1.33 (1.10, 1.60)	0.004
60–85	5.25 (4.47, 6.16)	<0.001	3.03 (2.52, 3.64)	<0.001
Gender				
Women	1.00		1.00	
Men	0.99 (0.87, 1.13)	0.906	0.93 (0.81, 1.08)	0.358
Marital status				
Never married	1.00			
Ever married	1.68 (1.39, 2.02)	<0.001		
Education attainment				
Elementary school or illiterate	1.00			
Junior high school	0.59 (0.46, 0.76)	<0.001		
Senior high school	0.60 (0.47, 0.76)	<0.001		
College/university or higher	0.56 (0.44, 0.70)	<0.001		
Occupation				
Employed	1.00		1.00	
Own business	1.10 (0.93, 1.31)	0.273	0.97 (0.80, 1.17)	0.766
Others	1.71 (1.45, 2.02)	<0.001	1.27 (1.05, 1.54)	0.013
Ability to pay for medication				
Very or fairly difficult	1.00		1.00	
Very or fairly easy	0.37 (0.32, 0.43)	<0.001	0.66 (0.56, 0.78)	<0.001
Social status				
Low	1.00		1.00	
Middle or high	0.74 (0.63, 0.87)	<0.001	1.16 (0.96, 1.40)	0.115
Lockdown measure				
No	1.00		1.00	
Yes	3.33 (2.91, 3.80)	<0.001	1.85 (1.56, 2.18)	<0.001
S-COVID-19-S^a^				
No	1.00		1.00	
Yes	4.14 (3.60, 4.75)	<0.001	2.40 (2.05, 2.81)	<0.001
BMI, kg/m^2^				
Underweight (BMI <18.5)	0.71 (0.55, 0.91)	0.008	0.78 (0.59, 1.02)	0.066
Normal weight (18.5 ≤ BMI <25.0)	1.00		1.00	
Overweight/obese (BMI ≥ 25.0)	0.97 (0.79, 1.19)	0.755	0.96 (0.76, 1.20)	0.709
Comorbidity				
None	1.00		1.00	
One	3.15 (2.70, 3.66)	<0.001	1.51 (1.26, 1.80)	<0.001
Two or more	7.61 (6.14, 9.45)	<0.001	2.19 (1.68, 2.85)	<0.001
Smoking^b^				
Never, stopped, or smoke less	1.00			
Unchanged or smoke more	0.98 (0.78, 1.23)	0.867		
Drinking alcohol^b^				
Never, stopped, or drink less	1.00			
Unchanged or drink more	1.04 (0.87, 1.25)	0.680		
Physical activity^b^				
Never, stopped, or exercise less	1.00		1.00	
Unchanged or exercise more	0.33 (0.29, 0.37)	<0.001	0.62 (0.53, 0.73)	<0.001
HL index, 1-score increment	0.95 (0.94, 0.96)	<0.001		
HES, 1-score increment	0.94 (0.93, 0.95)	<0.001	1.00 (0.99, 1.02)	0.687

PHQ, patient health questionnaire; OR, odds ratio; S-COVID-19-S, suspected corona virus disease-2019 symptoms; BMI, body mass index; HL, health literacy; HES, healthy eating score.

a The suspected COVID-19 symptoms including common symptom (fever, cough, dyspnea), less common symptom (myalgia, fatigue, sputum production, confusion, headache, sore throat, rhinorrhea, chest pain, hemoptysis, diarrhea, and nausea/vomiting).

b *People were asked whether their health-related behaviors are getting worse, better, or unchanged during COVID-19 pandemic as compared with those before the pandemic*.

### Mental Health Benefits of Healthy Dietary Intake

The results of interaction analysis showed that as compared with people who were not under the lockdown period and lowest HES, those who were under the lockdown period and lowest HES score had 10.6 times higher likelihood of depression (OR 10.60; 95% CI 6.88, 16.32; *p* < 0.001), whereas during the lockdown period, people with one score increment of HES resulted in 15% lower depression likelihood (OR 0.85; 95% CI 0.82, 0.89; *p* < 0.001; Table 5).

**Table 5:** Interactions of the lockdown measure and healthy dietary intake behavior on depression (*N* = 8,291).

**Interaction**	**Depression (PHQ ≥ 10)**			
	**Model 1**		**Model 2**	
	**OR (95% CI)**	***p***	**OR (95% CI)**	***p***
No lockdown and lowest HES	1.00		1.00	
Lockdown and lowest HES	30.51 (20.78, 44.80)	<0.001	10.60 (6.88, 16.32)	<0.001
No lockdown and HES, 1-score increment	1.05 (1.03, 1.07)	<0.001	1.06 (1.04, 1.08)	<0.001
Lockdown and HES, 1-score increment	0.81 (0.79, 0.84)	<0.001	0.85 (0.82, 0.89)	<0.001

*PHQ, patient health questionnaire; OR, odds ratio; HES, healthy eating score. Model 1: Interactions between the lockdown measure and healthy eating behavior on depression. Model 2: Adjusted for age, gender, occupation, ability to pay for medication, social status, suspected COVID-19 symptoms, body mass index, comorbidity, and physical activity*.

## Discussion

In the current study, people who were under the lockdown period had lower healthy dietary intake scores. This was similar to previous studies which illustrated that lockdown or home confinement measure negatively influenced dietary eating behaviors and habits (28–31, 54, 55). In addition, overweight and obese people ate less healthy than normal-weight individuals, which was found in the current study and previous studies (30). Besides, people with older age, being ever married, with S-COVID-19-S, comorbidity, and smoking and drinking behaviors also had worse dietary intake behavior. Social and environmental factors were found as determinants of eating behavior in a previous study (56). Therefore, nutrition support programs are important for vulnerable people to improve their dietary intake behavior (57, 58), especially during the pandemic and home confinement (30, 31).

Our study shows that people who were under the lockdown period had a higher likelihood of depression. Previous studies found that the proportion of psychological problems (e.g., depression, anxiety, and stress) has risen during the lockdown in general populations (27, 59) and in psychiatric patients (26). People with S-COVID-19-S had higher depression likelihood that was found in the current study and the previous one (45). In addition, people with older age and comorbidity were vulnerable to depression in the present study. The psychological consequence of COVID-19 pandemic was well-reported (53, 60), especially in the elderly (61, 62). Besides, people with underlying health conditions had a worse clinical course that was also reported (63, 64). Strategic mental health interventions are highly recommended to manage the psychological consequence of COVID-19 pandemic (65–68).

The most important finding of our study was that people with better healthy dietary intake behavior had lower depression likelihood during the lockdown period. This could be explained that better diet quality had benefits for lower risk of depression (34, 35). Anti-oxidant and anti-inflammatory nutrients from healthy foods can boost the immune function, reduce infection risk, and modulate the prognosis of COVID-19 disease (16, 17, 22, 23). In addition, depression has been protected and improved by doing the physical activity which was found in the current study and previous studies (69, 70). Furthermore, physical activity was linked to healthier eating behavior in the current study which further protects the people's mental health. Dietary intake and exercise was recognized as a key to healthy living (71). The findings provide important evidence to governments and organizations to develop strategic nutrition support programs to contain the pandemic and its adverse psychological consequences (21). HES-5 tool is suggested to use in clinical settings to quickly assess people' healthy eating behavior (38, 47), especially during the sensitive time of COVID-19 pandemic.

The current study shows that people with higher health literacy scores had a lower likelihood of depression. Health literacy has demonstrated an important role in evaluating online health information (72) in the digital world with diverse information and sources (73). Therefore, it is a critical skill for people during the COVID-19 pandemic and lockdown period. In addition, higher HL scores were independently associated with healthier behaviors (e.g., exercise, balanced diet) (74, 75) which further contribute to improve mental health (76). The policy-makers should be aware of and emphasize the roles and interplay between information providers and receivers which can improve people's understanding of medication information (77). Moreover, improving people's health literacy can help fight the infodemic and flatten the curve during the global health crisis (78, 79).

The current study has some limitations. First, research assistants and patients were vulnerable to virus infection during the pandemic. It was required to strictly follow the guidelines during the survey. Fortunately, researchers received great support from participating hospitals and health centers. In addition, there was no new case detected in the study settings during the data collection period (51). Second, the cross-sectional design with a non-random sample cannot generate a causal relationship. We have conducted the study on a large sample from 18 hospitals and health centers across Vietnam which can help in exploring the associations and interactions, and the findings can be cautiously generalized. Third, subjective measures with patients' self-reported information (e.g., height, weight) potentially bias the analysis. Therefore, findings should be interpreted with caution. Even though the HES-5 questionnaire was used for assessing the quality of the diet, and lack specificity, it is fast and easier than other validated questionnaires to measure healthy dietary intake, especially during the pandemic. Despite the mentioned limitations, findings of the current study substantially provide the evidence and direction for future research and practices to contain the COVID-19 disease and its related consequences.

## Conclusions

The COVID-19–induced lockdown or home confinement is a necessary measure to contain the viral infection. It shows negative impacts on dietary intake behavior and mental health. Fortunately, healthy dietary intake behavior can protect people's psychological health during the pandemic, especially during the lockdown period. The strategic public health approaches are required to develop nutritional support programs to improve the healthy eating behavior which further improves people's mental health and response to the pandemic.

## Data Availability Statement

The raw data supporting the conclusions of this article will be made available on reasonable request to the corresponding author.

## Ethics Statement

The study protocol was approved by each participating hospital, and the Institutional Ethical Review Committee of Hanoi School of Public Health, Vietnam (IRB No. 029/2020/YTCC-HD3 for the first stage from 14th February to 31st March 2020; and IRB No. 133/2020/YTCC-HD3 for the second stage from 1st April to 31st May 2020). The patients/participants provided their written informed consent to participate in this study.

## Author Contributions

KP and TVDu analyzed the data and drafted the article. KP, LP, DP, TT, HoaN, MN, HuuN, TH, HD, PN, MT, ThinD, HunN, TN, NN, CT, KT, TranD, LN, ThaoD, TV, BD, ThaiD, TP, TL, ND, HoaiN TM, DH, HuoN, KN, S-HY, JC, and TuyeD contributed to conceptualization, investigation, methodology, validation, writing review, and editing. KP, LP, DP, TT, HoaN, MN, HuuN, TH, HD, PN, MT, ThinD, HunN, TN, NN, CT, KT, TranD, LN, ThaoD, TV, BD, ThaiD, TP, TL, ND, HoaiN, TM, DH, HuoN, KN, and TuyeD conducted data curation. All authors contributed to the article and approved the submitted version.

## Conflict of Interest

The authors declare that the research was conducted in the absence of any commercial or financial relationships that could be construed as a potential conflict of interest.

## References

[1] Greenberg N Docherty M Gnanapragasam S Wessely S Managing mental health challenges faced by healthcare workers during covid-19 pandemic. BMJ. (2020) 368:m1211 10.1136/bmj.m1211 32217624

[2] Bassetti M Vena A Giacobbe DR. The novel Chinese coronavirus (2019-nCoV) infections: Challenges for fighting the storm. Eur J Clin Invest. (2020) 50:e13209. 10.1111/eci.13209 32003000 PMC7163647

[3] Phelan AL Katz R Gostin LO. The novel coronavirus originating in Wuhan, China: challenges for global health governance. JAMA. (2020) 323:709–10. 10.1001/jama.2020.1097 31999307

[4] Rubin R. The challenge of preventing COVID-19 spread in correctional facilities. JAMA. (2020) 323:1760–1. 10.1001/jama.2020.5427 32259189

[5] Xiang YT Jin Y Cheung T. Joint international collaboration to combat mental health challenges during the coronavirus disease 2019 pandemic. JAMA Psychiatry. (2020) 77:989–90. 10.1001/jamapsychiatry.2020.1057 32275289

[6] Campion J Javed A Sartorius N Marmot M. Addressing the public mental health challenge of COVID-19. Lancet Psychiatry. (2020) 7:657–9. 10.1016/S2215-0366(20)30240-6 32531299 PMC7282758

[7] Nicola M Alsafi Z Sohrabi C Kerwan A Al-Jabir A Iosifidis C. The socio-economic implications of the coronavirus pandemic (COVID-19): a review. Int J Surg. (2020) 78:185–93. 10.1016/j.ijsu.2020.04.018 32305533 PMC7162753

[8] Clark A Jit M Warren-Gash C Guthrie B Wang HHX Mercer SW. Global, regional, and national estimates of the population at increased risk of severe COVID-19 due to underlying health conditions in 2020: a modelling study. Lancet Glob Health. (2020) 8:e1003–17. 32553130 10.1016/S2214-109X(20)30264-3 PMC7295519

[9] Banerjee A Pasea L Harris S Gonzalez-Izquierdo A Torralbo A Shallcross L. Estimating excess 1-year mortality associated with the COVID-19 pandemic according to underlying conditions and age: a population-based cohort study. Lancet. (2020) 395:1715–25. 10.1016/S0140-6736(20)30854-0 32405103 PMC7217641

[10] World Health Organisation Coronavirus Disease (COVID-2019) Situation Reports. (2020). Available online at: https://www.who.int/emergencies/diseases/novel-coronavirus-2019/situation-reports/ (accessed March 05, 2020).

[11] Moradian N Ochs HD Sedikies C Hamblin MR Camargo CA Jr. The urgent need for integrated science to fight COVID-19 pandemic and beyond. J Transl Med. (2020) 18:205. 10.1186/s12967-020-02364-2 32430070 PMC7236639

[12] Holmes EA O'Connor RC Perry VH Tracey I Wessely S Arseneault L. Multidisciplinary research priorities for the COVID-19 pandemic: a call for action for mental health science. Lancet Psychiatry. (2020) 7:547–60. 10.1016/S2215-0366(20)30168-1 32304649 PMC7159850

[13] Nazir M Hussain I Tian J Akram S Mangenda Tshiaba S Mushtaq S. A multidimensional model of public health approaches against COVID-19. Int J Environ Res Public Health. (2020) 17:3780. 10.3390/ijerph17113780 32466581 PMC7312600

[14] Betsch C. How behavioural science data helps mitigate the COVID-19 crisis. Nat Hum Behav. (2020) 4:438. 10.1038/s41562-020-0866-1 32221514 PMC7101898

[15] Bavel JJV Baicker K Boggio PS Capraro V Cichocka A Cikara M. Using social and behavioural science to support COVID-19 pandemic response. Nat Hum Behav. (2020) 4:460–71. 10.1038/s41562-020-0884-z 32355299

[16] Gasmi A Noor S Tippairote T Dadar M Menzel A Bjørklund G. Individual risk management strategy and potential therapeutic options for the COVID-19 pandemic. Clin Immunol. (2020) 215:108409. 10.1016/j.clim.2020.108409 32276137 PMC7139252

[17] Zhang L Liu Y. Potential interventions for novel coronavirus in china: a systematic review. J Med Virol. (2020) 92:479–90. 10.1002/jmv.25707 32052466 PMC7166986

[18] Kakodkar P Kaka N Baig MN. A comprehensive literature review on the clinical presentation, and management of the pandemic coronavirus disease 2019 (COVID-19). Cureus. (2020) 12:e7560. 10.7759/cureus.7560 32269893 PMC7138423

[19] Morais AHA Aquino JS Silva-Maia JKD Vale SHL Maciel BLL Passos TS. Nutritional status, diet and viral respiratory infections: perspectives for SARS-CoV-2. Br J Nutr. (2020) 1–12. 10.1017/S0007114520003311. [Epub ahead of print]. 32843118 PMC7542326

[20] Fan Y Zhang Y Tariq A Jiang X Ahamd Z Zhihao Z. Food as medicine: a possible preventive measure against coronavirus disease (COVID-19). Phytother Res. (2020). 10.1002/ptr.6770. [Epub ahead of print]. 32468635 PMC7283886

[21] Zabetakis I Lordan R Norton C Tsoupras A. COVID-19: the inflammation link and the role of nutrition in potential mitigation. Nutrients. (2020) 12:1466. 10.3390/nu12051466 32438620 PMC7284818

[22] Calder PC Carr AC Gombart AF Eggersdorfer M Optimal nutritional status for a well-functioning immune system is an important factor to protect against viral infections. Nutrients. (2020) 12:1181 10.3390/nu12041181 PMC7469053 32756516

[23] Iddir M Brito A Dingeo G Fernandez Del Campo SS Samouda H La Frano MR. Strengthening the immune system and reducing inflammation and oxidative stress through diet and nutrition: considerations during the COVID-19 crisis. Nutrients. (2020) 12:1562. 10.3390/nu12061562 32471251 PMC7352291

[24] Vietnam Prime Minister PM Orders Strict Nationwide Social Distancing Rules, Starting April 1. (2020). Available online at: https://vietnamlawmagazine.vn/pm-orders-strict-nationwide-social-distancing-rules-starting-april-1-27108.html (accessed March 31, 2020).

[25] Lippi G Henry BM Bovo C Sanchis-Gomar F. Health risks and potential remedies during prolonged lockdowns for coronavirus disease 2019 COVID-19. Diagnosis. (2020) 7:85–90. 10.1515/dx-2020-0041 32267243

[26] Hao F Tan W Jiang L Zhang L Zhao X Zou Y. Do psychiatric patients experience more psychiatric symptoms during COVID-19 pandemic and lockdown? A case-control study with service and research implications for immunopsychiatry. Brain Behav Immun. (2020) 87:100–6. 10.1016/j.bbi.2020.04.069 32353518 PMC7184991

[27] Ozamiz-Etxebarria N Idoiaga Mondragon N Dosil Santamaría M Picaza Gorrotxategi M Psychological symptoms during the two stages of lockdown in response to the COVID-19 outbreak: an investigation in a sample of citizens in Northern Spain. Front Psychol. (2020) 11:1491 10.3389/fpsyg.2020.02116 32625157 PMC7314923

[28] Di Renzo L Gualtieri P Pivari F Soldati L Attinà A Cinelli G. Eating habits and lifestyle changes during COVID-19 lockdown: an Italian survey. J Transl Med. (2020) 18:229. 10.1186/s12967-020-02399-5 32513197 PMC7278251

[29] Ammar A Brach M Trabelsi K Chtourou H Boukhris O Masmoudi L. Effects of COVID-19 home confinement on eating behaviour and physical activity: results of the ECLB-COVID19 International Online Survey. Nutrients. (2020) 12:1583. 10.3390/nu12061583 32481594 PMC7352706

[30] Sidor A Rzymski P. Dietary choices and habits during COVID-19 lockdown: experience from Poland. Nutrients. (2020) 12:1657. 10.3390/nu12061657 32503173 PMC7352682

[31] Rodríguez-Pérez C Molina-Montes E Verardo V Artacho R García-Villanova B Guerra-Hernández EJ. Changes in dietary behaviours during the COVID-19 outbreak confinement in the Spanish COVIDiet study. Nutrients. (2020) 12:1730. 10.3390/nu12061730 32531892 PMC7353108

[32] Martinez-Ferran M de la Guía-Galipienso F Sanchis-Gomar F Pareja-Galeano H. Metabolic impacts of confinement during the COVID-19 pandemic due to modified diet and physical activity habits. Nutrients. (2020) 12:1549. 10.3390/nu12061549 32466598 PMC7352228

[33] Butler MJ Barrientos RM. The impact of nutrition on COVID-19 susceptibility and long-term consequences. Brain Behav Immun. (2020) 87:53–4. 10.1016/j.bbi.2020.04.040 32311498 PMC7165103

[34] Molendijk M Molero P Ortuño Sánchez-Pedreño F Van der Does W Angel Martínez-González M. Diet quality and depression risk: a systematic review and dose-response meta-analysis of prospective studies. J Affect Disord. (2018) 226:346–54. 10.1016/j.jad.2017.09.022 29031185

[35] Li Y Lv M-R Wei Y-J Sun L Zhang J-X Zhang H-G. Dietary patterns and depression risk: a meta-analysis. Psychiatry Res. (2017) 253:373–82. 10.1016/j.psychres.2017.04.020 28431261

[36] Gasmi A Tippairote T Mujawdiya PK Peana M Menzel A Dadar M. Micronutrients as immunomodulatory tools for COVID-19 management. Clin Immunol. (2020) 220:108545. 10.1016/j.clim.2020.108545 32710937 PMC7833875

[37] Batlle-Bayer L Aldaco R Bala A Puig R Laso J Margallo M. Environmental and nutritional impacts of dietary changes in Spain during the COVID-19 lockdown. Sci Total Environ. (2020) 748:141410. 10.1016/j.scitotenv.2020.141410 32798877 PMC7395635

[38] Shams-White MM Chui K Deuster PA McKeown NM Must A. Investigating items to improve the validity of the five-item healthy eating score compared with the 2015 healthy eating index in a military population. Nutrients. (2019) 11:251. 10.3390/nu11020251 30678099 PMC6412234

[39] Prime Minister of Vietnam Gov't Extends Social Distancing for at Least One Week in 28 Localities. (2020). Available online at: http://news.chinhphu.vn/Home/Govt-extends-social-distancing-for-at-least-one-week-in-28-localities/20204/39735.vgp (accessed April 15, 2020).

[40] Editorial Team Overview of Novel Coronavirus (2019-nCoV). (2020). Available online at: https://bestpractice.bmj.com/topics/en-gb/3000165 (accessed February 10, 2020).

[41] Quan H Li B Couris CM Fushimi K Graham P Hider P. Updating and validating the Charlson comorbidity index and score for risk adjustment in hospital discharge abstracts using data from 6 countries. Am J Epidemiol. (2011) 173:676–82. 10.1093/aje/kwq433 21330339

[42] Duong TV Aringazina A Baisunova G Nurjanah N Pham TV Pham KM. Development and validation of a new short-form health literacy instrument (HLS-SF12) for the general public in six Asian countries. Health Lit Res Pract. (2019) 3:e91–102. 10.3928/24748307-20190225-01 31294310 PMC6607763

[43] Duong TV Nguyen TTP Pham KM Nguyen KT Giap MH Tran TDX. Validation of the short-form health literacy questionnaire (HLS-SF12) and its determinants among people living in rural areas in Vietnam. Int J Environ Res Public Health. (2019) 16:3346. 10.3390/ijerph16183346 31514271 PMC6765800

[44] Ho HV Hoang GT Pham VT Duong TV Pham KM. Factors associated with health literacy among the elderly people in Vietnam. Biomed Res Int. (2020) 2020:3490635. 10.1155/2020/3490635 32309429 PMC7139882

[45] Nguyen HC Nguyen MH Do BN Tran CQ Nguyen TTP Pham KM. People with suspected COVID-19 symptoms were more likely depressed and had lower health-related quality of life: The potential benefit of health literacy. J Clin Med. (2020) 9:965. 10.3390/jcm9040965 32244415 PMC7231234

[46] Nguyen HT Do BN Pham KM Kim GB Dam HTB Nguyen TT. Fear of COVID-19 scale—associations of its scores with health literacy and health-related behaviors among medical students. Int J Environ Res Public Health. (2020) 17:4164. 10.3390/ijerph17114164 32545240 PMC7311979

[47] Purvis DL Lentino CV Jackson TK Murphy KJ Deuster PA. Nutrition as a component of the performance triad: how healthy eating behaviors contribute to soldier performance and military readiness. US Army Med Dep J. (2013) 66–78. 24146244

[48] Kroenke K Spitzer RL Williams JBW. The PHQ-9: validity of a brief depression severity measure. J Gen Intern Med. (2001) 16:606–13. 10.1046/j.1525-1497.2001.016009606.x 11556941 PMC1495268

[49] National Center for Immunization and Respiratory Diseases (NCIRD) Division of Viral Diseases What Healthcare Personnel Should Know About Caring for Patients With Confirmed or Possible 2019-nCoV Infection. (2020). Available online at: https://www.cdc.gov/coronavirus/2019-ncov/hcp/caring-for-patients.html (accessed February 07, 2020).

[50] World Health Organization (WHO) Country & Technical Guidance - Coronavirus Disease (COVID-19). (2020). Available online at: https://www.who.int/emergencies/diseases/novel-coronavirus-2019/technical-guidance (accessed February 10, 2020).

[51] Ministry of Health Coronavirus Disease (COVID-19) Outbreak in Vietnam. (2020). Available online at: https://ncov.moh.gov.vn/ (accessed April 05, 2020).

[52] Maldonado G Greenland S. Simulation study of confounder-selection strategies. Am J Epidemiol. (1993) 138:923–36. 10.1093/oxfordjournals.aje.a116813 8256780

[53] Vindegaard N Eriksen Benros M. COVID-19 pandemic and mental health consequences: systematic review of the current evidence. Brain Behav Immun. (2020) 89:531–42. 10.1016/j.bbi.2020.05.048 32485289 PMC7260522

[54] Di Renzo L Gualtieri P Cinelli G Bigioni G Soldati L Attinà A Psychological aspects and eating habits during COVID-19 home confinement: results of EHLC-COVID-19 Italian Online Survey. Nutrients. (2020) 12:2152 10.3390/nu12072152 PMC7401000 32707724

[55] Cancello R Soranna D Zambra G Zambon A Invitti C. Determinants of the lifestyle changes during COVID-19 pandemic in the residents of Northern Italy. Int J Environ Res Public Health. (2020) 17:6287. 10.3390/ijerph17176287 32872336 PMC7504331

[56] Marcone MF Madan P Grodzinski B. An overview of the sociological and environmental factors influencing eating food behavior in Canada. Front Nutr. (2020) 7:77. 10.3389/fnut.2020.00077 32582753 PMC7283517

[57] Liu Y-H Gao X Mitchell DC Wood GC Still CD Jensen GL. Diet quality is associated with mortality in adults aged 80 years and older: a prospective study. J Am Geriatr Soc. (2019) 67:2180–5. 10.1111/jgs.16089 31386173

[58] Willett W Rockström J Loken B Springmann M Lang T Vermeulen S. Food in the anthropocene: the EAT–lancet commission on healthy diets from sustainable food systems. Lancet. (2019) 393:447–92. 10.1016/S0140-6736(18)31788-4 30660336

[59] Odriozola-González P Planchuelo-Gómez Á Irurtia MJ de Luis-García R. Psychological effects of the COVID-19 outbreak and lockdown among students and workers of a Spanish university. Psychiatry Res. (2020) 290:113108. 10.1016/j.psychres.2020.113108 32450409 PMC7236679

[60] González-Sanguino C Ausín B Castellanos M Saiz J López-Gómez A Ugidos C. Mental health consequences during the initial stage of the 2020 Coronavirus pandemic (COVID-19) in Spain. Brain Behav Immun. (2020) 87:172–6. 10.1016/j.bbi.2020.05.040 32405150 PMC7219372

[61] Meng H Xu Y Dai J Zhang Y Liu B Yang H The psychological effect of COVID-19 on the elderly in China. Psychiatry Res. (2020) 289:112983 10.1016/j.psychres.2020.112983 PMC7151427 32388175

[62] Armitage R Nellums LB. COVID-19 and the consequences of isolating the elderly. Lancet Public Health. (2020) 5:e256. 10.1016/S2468-2667(20)30061-X 32199471 PMC7104160

[63] Wang B Li R Lu Z Huang Y. Does comorbidity increase the risk of patients with COVID-19: evidence from meta-analysis. Aging. (2020) 12:6049–57. 10.18632/aging.103000 32267833 PMC7185114

[64] Guan WJ Liang WH Zhao Y Liang HR Chen ZS Li YM. Comorbidity and its impact on 1590 patients with COVID-19 in China: a nationwide analysis. Eur Respir J. (2020) 55:2000547. 10.1183/13993003.01227-2020 32217650 PMC7098485

[65] Duan L Zhu G. Psychological interventions for people affected by the COVID-19 epidemic. Lancet Psychiatry. (2020) 7:300–2. 10.1016/S2215-0366(20)30073-0 32085840 PMC7128328

[66] Xiang Y-T Yang Y Li W Zhang L Zhang Q Cheung T. Timely mental health care for the 2019 novel coronavirus outbreak is urgently needed. Lancet Psychiatry. (2020) 7:228–9. 10.1016/S2215-0366(20)30046-8 32032543 PMC7128153

[67] Liu S Yang L Zhang C Xiang Y-T Liu Z Hu S. Online mental health services in China during the COVID-19 outbreak. Lancet Psychiatry. (2020) 7:e17–8. 10.1016/S2215-0366(20)30077-8 32085841 PMC7129099

[68] Galea S Merchant RM Lurie N. The mental health consequences of COVID-19 and physical distancing: the need for prevention and early intervention. JAMA Intern Med. (2020) 180:817–8. 10.1001/jamainternmed.2020.1562 32275292

[69] Mammen G Faulkner G. Physical activity and the prevention of depression: a systematic review of prospective studies. Am J Prev Med. (2013) 45:649–57. 10.1016/j.amepre.2013.08.001 24139780

[70] Kvam S Kleppe CL Nordhus IH Hovland A. Exercise as a treatment for depression: a meta-analysis. J Affect Disord. (2016) 202:67–86. 10.1016/j.jad.2016.03.063 27253219

[71] Burd NA McKenna CF Salvador AF Paulussen KJM Moore DR. Dietary protein quantity, quality, and exercise are key to healthy living: a muscle-centric perspective across the lifespan. Front Nutr. (2019) 6:83. 10.3389/fnut.2019.00083 31245378 PMC6563776

[72] Diviani N van den Putte B Giani S van Weert JC. Low health literacy and evaluation of online health information: a systematic review of the literature. J Med Internet Res. (2015) 17:e112. 10.2196/jmir.4018 25953147 PMC4468598

[73] Norman CD Skinner HA. eHealth literacy: Essential skills for consumer health in a networked world. J Med Internet Res. (2006) 8:e9. 10.2196/jmir.8.2.e9 16867972 PMC1550701

[74] Mitsutake S Shibata A Ishii K Oka K. Associations of eHealth literacy with health behavior among adult internet users. J Med Internet Res. (2016) 18:e192. 10.2196/jmir.5413 27432783 PMC4969548

[75] Duong TV Chiu C-H Lin C-Y Wong T-C Chen Y-C Chang PW. E-healthy diet literacy scale and its relationship with behaviors and health outcomes in Taiwan. Health Promot Int. (2020) daaa033. 10.1093/heapro/daaa033. [Epub ahead of print]. 32267935

[76] Owen L Corfe B. The role of diet and nutrition on mental health and wellbeing. Proc Nutr Soc. (2017) 76:425–6. 10.1017/S0029665117001057 28707609

[77] Roshi D Burazeri G Schröder-Bäck P Toçi E Italia S Ylli A. Understanding of medication information in primary health care: a cross-sectional study in a South Eastern European population. Front Public Health. (2020) 8:388. 10.3389/fpubh.2020.00388 32903804 PMC7438893

[78] Chong YY Cheng HY Chan HYL Chien WT Wong SYS. COVID-19 pandemic, infodemic and the role of eHealth literacy. Int J Nurs Stud. (2020) 108:103644. 10.1016/j.ijnurstu.2020.103644 32447127 PMC7255119

[79] Košir U Sørensen K. COVID-19: the key to flattening the curve is health literacy. Perspect Public Health. (2020) 1757913920936717. 10.1177/1757913920936717 . [Epub ahead of print]. 32650704

