# Supplementary material for: Healthy Dietary Intake Behavior Potentially Modifies the Negative Effect of COVID-19 Lockdown on Depression: A Hospital and Health Center Survey
Source: Front Nutr. 2020 Nov 16;7:581043. doi: 10.3389/fnut.2020.581043 (PMC7701254; doi:10.3389/fnut.2020.581043)
Supplement: Supplementary file 1 [file Table_1.DOCX]

**Supplementary Table 1 |** Construct and convergent validity, internal consistency, floor and ceiling effects the healthy eating score (*N* =8291).

| **Items** | **HES** |
| --- | --- |
| 1. Fruits | 0.75 |
| 1. Vegetables | 0.71 |
| 1. Whole grains | 0.70 |
| 1. Dairy | 0.73 |
| 1. Fish | 0.63 |
| Percentage of variance, % | 49.43 |
| Item-scale convergent validity, mean of Rho (range) | 0.68 (0.57-0.73) |
| Internal consistency, Cronbach’s alpha | 0.74 |
| Floor effects, % | 0.30 |
| Ceiling effect, % | 1.00 |

*HES, healthy eating score; Rho, Spearman’s correlation coefficient.*

**Supplementary Table 2 |** Spearman’s correlations of studied variables (*N* =8291).

|  | **Age** | **Gender** | **Marital status** | **Education** | **Occupation** | **Ability to pay for medication** | **Social status** | **Lockdown measure** | **S-COVID-19-S** | **BMI** | **Comorbidity** | **Smoking** | **Drinking** | **Physical activity** | **HL index** | **HES** |
| --- | --- | --- | --- | --- | --- | --- | --- | --- | --- | --- | --- | --- | --- | --- | --- | --- |
| Gender | 0.04 |  |  |  |  |  |  |  |  |  |  |  |  |  |  |  |
| Marital status | 0.38 | -0.06 |  |  |  |  |  |  |  |  |  |  |  |  |  |  |
| Education | -0.42 | 0.04 | -0.27 |  |  |  |  |  |  |  |  |  |  |  |  |  |
| Occupation | 0.10 | -0.07 | -0.16 | -0.26 |  |  |  |  |  |  |  |  |  |  |  |  |
| Ability to pay for medication | -0.17 | -0.01 | -0.08 | 0.19 | -0.17 |  |  |  |  |  |  |  |  |  |  |  |
| Social status | -0.10 | 0.02 | -0.01 | 0.22 | -0.14 | 0.29 |  |  |  |  |  |  |  |  |  |  |
| Lockdown measure | 0.06 | 0.01 | 0.12 | 0.01 | 0.00 | -0.20 | -0.10 |  |  |  |  |  |  |  |  |  |
| S-COVID-19-S | 0.21 | 0.00 | 0.13 | 0.00 | -0.03 | -0.16 | -0.04 | 0.13 |  |  |  |  |  |  |  |  |
| BMI | 0.05 | 0.06 | 0.13 | -0.01 | -0.08 | 0.03 | 0.04 | 0.01 | -0.01 |  |  |  |  |  |  |  |
| Comorbidity | 0.31 | 0.03 | 0.13 | -0.07 | -0.01 | -0.13 | -0.08 | 0.25 | 0.31 | 0.02 |  |  |  |  |  |  |
| Smoking | 0.04 | 0.24 | 0.04 | -0.08 | -0.01 | -0.04 | -0.06 | -0.05 | -0.02 | 0.02 | -0.02 |  |  |  |  |  |
| Drinking | -0.01 | 0.27 | -0.04 | 0.00 | -0.01 | 0.03 | 0.00 | -0.17 | -0.04 | 0.02 | -0.08 | 0.45 |  |  |  |  |
| Physical activity | -0.06 | 0.06 | -0.10 | -0.02 | -0.01 | 0.18 | 0.08 | -0.38 | -0.19 | -0.04 | -0.19 | 0.11 | 0.21 |  |  |  |
| HL index | -0.32 | 0.04 | -0.19 | 0.24 | -0.06 | 0.25 | 0.22 | -0.12 | -0.29 | -0.01 | -0.31 | -0.06 | 0.02 | 0.22 |  |  |
| HES | -0.13 | -0.04 | -0.05 | 0.11 | -0.01 | 0.11 | 0.11 | -0.16 | -0.17 | -0.03 | -0.26 | -0.10 | -0.07 | 0.07 | 0.23 |  |
| PHQ | 0.22 | 0.00 | 0.06 | -0.03 | 0.07 | -0.15 | -0.04 | 0.20 | 0.23 | 0.02 | 0.24 | 0.00 | 0.00 | -0.19 | -0.17 | -0.11 |

*S-COVID-19-S, suspected corona virus disease-2019 symptoms; BMI, body mass index; HL, health literacy; HES, healthy eating score; PHQ, patient health questionnaire.*
